# Antibiotic Prophylaxis in Maxillofacial Surgery and Evidence-Based Practice: A Systematic Review

**DOI:** 10.7759/cureus.95634

**Published:** 2025-10-28

**Authors:** Gayatri Moharana, Rajat Mohanty, Spandita Das

**Affiliations:** 1 Department of Oral and Maxillofacial Surgery, Kalinga Institute of Dental Sciences (KIDS) Kalinga Institute of Industrial Technology (KIIT) Deemed to be University, Bhubaneswar, IND; 2 Department of Public Health Dentistry, Kalinga Institute of Dental Sciences (KIDS) Kalinga Institute of Industrial Technology (KIIT) Deemed to be University, Bhubaneswar, IND

**Keywords:** antibiotic prophylaxis, dental implants, fracture, third molar, tooth extraction

## Abstract

Antibiotic prophylaxis is defined as the intentional administration of antibiotics with the goal of preventing infection prior to the occurrence of contamination by surgical incision. So, it can either be given before the procedure or after the surgery to prevent infection. The aim of this study was to review the current literature and evaluate whether the use of preoperative, perioperative, or postoperative antibiotic prophylaxis influences outcomes in patients undergoing oral and maxillofacial surgeries. This systematic review was conducted in line with the Preferred Reporting Items for Systematic Reviews and Meta-Analyses (PRISMA) guidelines and was registered in the International Prospective Register of Systematic Reviews (PROSPERO). A comprehensive search of PubMed, Scopus, and Web of Science was conducted for studies published up to May 2025, utilizing Medical Subject Headings (MeSH) terms based on the Population, Intervention, Comparison, and Outcome (PICO) framework. Eligible studies included randomized controlled trials (RCTs) and cohort studies involving patients undergoing maxillofacial procedures such as orthognathic surgery, trauma surgery, dental implant placement, tumor resection, and temporomandibular joint surgery. The outcomes assessed were surgical site infections (SSIs), wound infections, abscess formation, antibiotic-related side effects, antimicrobial resistance, length of hospital stay, and cost-effectiveness. Thirteen studies met the inclusion criteria and were categorized under five clinical domains: dental implants, surgical tooth extraction, maxillofacial trauma, orthognathic surgery, and drug allergy. The Newcastle-Ottawa Scale (NOS) was applied to evaluate potential sources of bias across the included studies. Overall, the included studies were of fair quality, supporting a moderate level of confidence in the conclusions drawn. Prophylactic antibiotics in maxillofacial surgery show limited overall benefit, with selective advantages in complex or high-risk cases. Routine use is not consistently justified, highlighting the need for judicious application and further high-quality research to guide practice.

## Introduction and background

Antibiotic prophylaxis is defined as the intentional administration of antibiotics with the goal of preventing infection, prior to the occurrence of contamination by surgical incision. So, it can either be given before the procedure or after the surgery to prevent infection. Surgical site infections (SSIs) may result in substantial mortality and morbidity after surgery. The length of the procedure, the type of wound, and the patient's American Society of Anesthesiologists (ASA) score all affect the chance of SSIs. Antibiotic prophylaxis is well recognized to be indicated in cases when there is an increased risk of SSIs [1].

Preventive antibiotic treatment is believed to be crucial in promoting the best possible recovery following surgery. When surgical damage occurs on the skin, the primary physical barrier that prevents microbes from entering the body is destroyed. This occurrence allows the bacteria to infiltrate deep tissues, where they may proliferate and cause infection. The likelihood of infection increases with the amount of bacteria present, as well as if the procedure is clean, clean-contaminated, contaminated, or dirty. The risk of postsurgical infection increases with the level of infection in the wound [2].

The variation of the operation has a significant impact on the prevalence of SSIs. The American Society of Health-System Pharmacists (ASHP) classifies surgery into four categories, each of which is further described in the context of this study, with typical examples of procedures in oral and maxillofacial surgery [3]. Among them, the first is clean (e.g., atraumatic procedures or where neither the gastrointestinal, genitourinary, nor respiratory tracts are involved, such as cervical lymph node excisions). The second is clean-contaminated (e.g., procedures that involve the gastrointestinal or respiratory tract, such as third molar removal). The third is contaminated (e.g., surgery in acute inflammatory conditions, like open mandible fracture repair with osteosynthesis). And the fourth one is dirty (e.g., procedures involving pus or compound/open injuries such as odontogenic abscess).

The aim of the study was to systematically review the current literature and examine whether the use of preoperative, perioperative, or postoperative antibiotic prophylaxis in patients undergoing oral and maxillofacial surgeries. The study also aimed to identify evidence-based antibiotic regimes for use in oral and maxillofacial surgery.

## Review

Methods

The current review was conducted in accordance with the guidelines of Preferred Reporting Items for Systematic Reviews and Meta-analyses (PRISMA) [3]. The study protocol was registered in the International Prospective Register of Systematic Reviews (PROSPERO) database (Registration ID: CRD42023456441). The review was based on the pre-specified question; the search strategy was implemented using pertinent Medical Subject Headings (MeSH) terms in accordance with the Population, Intervention, Comparison, and Outcome (PICO) model.

Inclusion and Exclusion Criteria

This study included randomized controlled trials (RCTs) and cohort studies involving patients undergoing maxillofacial surgeries such as orthognathic surgery, trauma surgery, implant placement, tumor resection, and temporomandibular joint (TMJ) surgery; studies evaluating antibiotic prophylaxis (preoperative, perioperative, or postoperative) compared to no antibiotics, different antibiotic regimens, or varying durations of antibiotic use; and studies reporting SSI rates, postoperative complications, adverse effects, and antimicrobial resistance, published in peer-reviewed journals in English within the last 15 to 20 years. Exclusion criteria comprised of case reports, case series, narrative reviews, and studies focusing on non-maxillofacial surgeries or patients with pre-existing infections or immunocompromised conditions; studies where antibiotics are used for therapeutic treatment rather than prophylaxis, or those that do not report relevant infection rates or postoperative complications; non-peer-reviewed studies, conference abstracts, preprints, or articles published in languages other than English. Studies without full-text access, unpublished works, and articles not relevant to the topic were excluded from this review. 

PICO for Systematic Review on Antibiotic Prophylaxis in Maxillofacial Surgery

P: Patients undergoing maxillofacial surgery (e.g., orthognathic surgery, trauma surgery, dental implant surgery, tumor resection, TMJ surgery). I: Use of antibiotic prophylaxis (preoperative, perioperative, or postoperative), including different classes, dosages, and durations of antibiotics. C: No antibiotic prophylaxis or different antibiotic regimens (e.g., single-dose vs. multiple-dose, short-course vs. long-course, different antibiotic classes). O: The primary outcomes were incidence of SSIs, wound infections, and abscess formation; and the secondary outcomes were antibiotic-related adverse effects, antimicrobial resistance, length of hospital stay, and cost-effectiveness.

Information Sources, Search, and Study Selection

Articles from three electronic databases, PubMed, Scopus, and Web of Science, until May 2025, were searched, as they together provide broad and overlapping coverage of the biomedical, public health, and dental research domains relevant to this review. The articles were identified according to MeSH terms, which were searched from PubMed using Boolean operators (AND, OR, NOT) to combine searches and entry terms for Scopus and Web of Science (Table 1).

**Table 1:** Search strategy

Database	Search String
Pubmed	Antibiotic Prophylaxis"[MeSH Terms] AND ("surgery, oral"[MeSH Terms] OR "Oral Surgical Procedures"[MeSH Terms])
Scopus	("Antibiotic Prophylaxis") AND ("Surgery, Oral" OR "Oral Surgical Procedures")
Web of Science	("Antibiotic Prophylaxis") AND ("Surgery, Oral" OR "Oral Surgical Procedures")

Data Extraction: Selection and Coding

The primary review authors independently screened the titles and abstracts of the literature search results together with an assigned co-screener, who provided supervisory oversight and validated the selection process. Disagreements were resolved through discussions, and the final decision was made by either party. The full-text articles were then independently screened for inclusion and further processing. As needed, corresponding authors were contacted to provide the missing information. A distinctively customized Microsoft Excel sheet (Microsoft Corporation, Redmond, WA) was fabricated to register the data obtained from the studies. The data extraction sheet was independently used by the two reviewers to capture the following data items for each of the articles included in this review.

A meta-analysis was not performed due to substantial heterogeneity among the included studies in terms of study design, outcome measures, and reporting formats, which precluded quantitative synthesis.

Results

Study Selection Results

**Figure 1:**
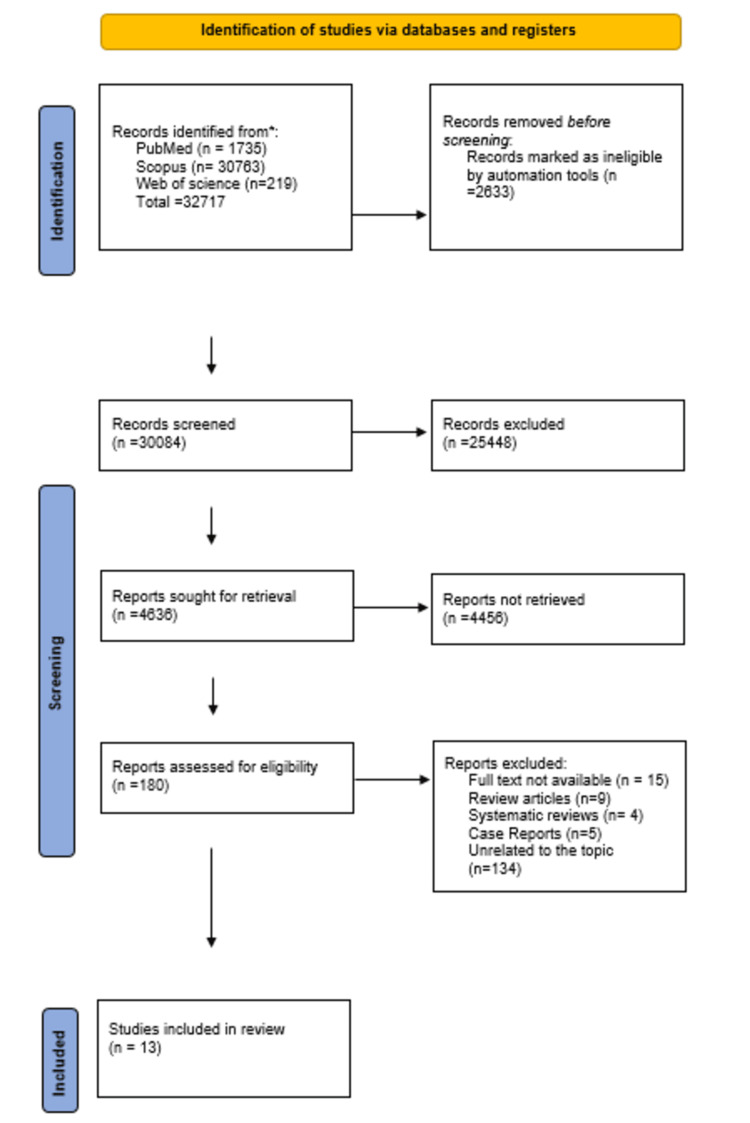
A PRISMA flowchart outlining the study selection process PRISMA: Preferred Reporting Items for Systematic Reviews and Meta-Analyses

As shown in the PRISMA chart, our initial search yielded 32,717 potentially eligible citations. After removing duplicates and eliminating citations based on title and abstract, and full-text review, we identified 13 studies that met our inclusion criteria. The articles were subcategorized into five headings, namely, dental implants, surgical tooth extraction, maxillofacial trauma, orthognathic surgery, and drug allergy. Figure 1 depicts the selection criteria based on PRISMA guidelines [3]. 

Study Feature

The fundamental features of the incorporated articles are summarized in Table 2. The incorporated articles were printed in a good, reputable journal indexed in the Web of Science, Scopus, and PubMed. The majority of the studies were published in the Journal of Cranio-Maxillo-Facial Surgery, Clinical Oral Implants Research, Medicina Oral Patologia, Oral Cirugia Bucal, Oral Surgery, Oral Medicine, Oral Pathology, Oral Radiology, Journal of Oral and Maxillofacial Surgery, and International Journal of Oral and Maxillofacial Surgery.

Table 2 documents the characteristics of the included studies that were assessed in this systematic review, including the study design, sample size, and follow-up duration [4-16]. Two independent reviewers assessed the studies, and any disagreements were resolved through discussion or consultation with a third reviewer. Systematic evaluation of study characteristics and risk of bias ensured the reliability and validity of the review's findings.

**Table 2:** Characteristics of the included studies VAS: Visual Analog Scale; RR: risk ratio; NNTB: number needed to treat for benefit; BRONJ: bisphosphonate-related osteonecrosis of the jaw; BP: blood pressure; SSIs: surgical site infections; CRP: C-reactive protein; HR: hazard ratio

Sl. No.	Authors	Place of Study	Study Design	Sample Size	Intervention	Comparator	Outcome Measures	Conclusion
Dental implants								
1	Tabrizi et al. [4]	Iran	Randomized controlled trial	450	Different antibiotic regimens	Placebo or standard care	Postoperative infection rate, implant failure	No significant difference in infection across groups (p = 0.62). Implant failure: 2 in group 2, 1 in group 3.
2	Tan et al. [5]	Spain	Randomized controlled trial	329	Different flap management strategies	Negative control group	VAS pain scores, post-surgical complications, and wound closure at week 4	No major differences in pain or complications; significantly better wound closure in test groups (P = 0.01).
3	Nolan et al. [6]	Ireland	Randomized controlled trial	55	Prophylactic antibiotics before implant surgery	No antibiotics	Implant survival rate, postoperative pain, and discomfort in implant failure cases	Antibiotics increased implant survival (100% vs. 82%) and reduced pain. Implant failure is associated with more discomfort (P = 0.003).
Surgical tooth extraction								
4	Yanine et al.[7]	Chile	Randomized controlled trial	154	Experimental group (unspecified intervention)	Control group	Postoperative infections, rescue analgesic use. RR for infection = 0.4 (95% CI: 0.08–1.99, p = 0.41). RR for analgesic use = 0.49 (95% CI: 0.32–0.75, p < 0.05)	No significant difference in infection rates; significantly lower rescue analgesia use in the experimental group (NNTB = 3).
5	Mariscal-Cazalla et al. [8]	Spain	Randomized controlled trial	92	Three treatment groups (types not specified)	Groups 1 & 2 vs. group 3	Pain and inflammation intensity at 48 hours, 72 hours, and one week; rescue medication usage	Group 3 had significantly higher pain and medication use (P < 0.05, P = 0.013).
6	Bodem et al. [9^]^	Germany	Cohort study	61	Ongoing IV bisphosphonate therapy	Completed or stopped therapy	Risk of BRONJ, influence of IV BP duration, feeding tube use, and antimicrobial prophylaxis	No significant association found with BRONJ development for any parameter studied.
7	Arteagoitia et al. [10]	Spain	Randomized controlled trial	118	Prophylactic antibiotics	Placebo	Postoperative infection occurrence within one week and after one week	No statistically significant difference in infection rates between groups. Five infections in the placebo group vs. two in the antibiotic group.
Oral and maxillofacial trauma								
8	Gaal et al. [11]	United States of America	Cohort study	510	Study group vs. control group	58 (study) vs. 452 (control)	SSIs, adverse events, risk factors	SSI not significantly different (p=0.13). Only smoking was significantly associated with higher SSI risk
Orthognathic surgery								
9	Ghantous et al. [12]	Israel	Randomized controlled trial	78	Prophylactic antibiotic/placebo	Placebo	Postoperative temperature, CRP, infection rate, and surgical discharge	No difference in infection rate. Two serous discharges in the placebo group; one required surgery.
10	Davis et al. [13]	Canada	Cohort study	2521	Cefazolin, penicillin, clindamycin	Comparison of antibiotics	Postoperative infection rate	Cefazolin group had significantly lower infection than penicillin and clindamycin groups (P < .0001).
Miscellaneous studies								
11	Roistacher et al. [14]	United States of America	Cohort study	2058	Patients with vs. without penicillin allergy	-	SSI incidence, adjusted hazard ratio	Penicillin allergy associated with a higher SSI risk (HR=2.61); the effect mediated by alternative antibiotic selection.
12	Marimuthu et al.[15]	India	Randomized controlled trial	75	Two different prophylaxis regimens	Group I vs. Group II	Postoperative infection, surgical wound splitting	No infection in group I; one infection in group II (p=1.000). No wound split observed in either group.
13	Wahab et al. [16]	India	Randomized controlled trial	60	Short-course antibiotics vs. single dose	Short course vs. single dose	Need for rescue antibiotics	Six patients in the single-dose group vs. 1 in short short-course group needed rescue antibiotics—favoring short-course.

Each study was evaluated using the tool based on eight criteria that are divided into three areas: choosing the study groups, assessing the groups' comparability, and determining the exposure or outcome of interest for case-control or cohort studies, respectively. Each quality item was given a star, which acts as a rapid visual evaluation. The best caliber research received up to nine stars in the star system. The formal risk of bias assessment tool, the Newcastle-Ottawa Scale (NOS), was used to evaluate potential biases [17]. The University of Newcastle in Australia and the University of Ottawa in Canada collaborated to develop the approach, which defined variables for data extraction through a Delphi process. After that, the scale was improved and tested using systematic reviews. Tools specifically designed for cohort and case-control studies were created. Additionally, it has been modified for ubiquity. The maximum score of 6 was by one study, and a score of 5 was by three studies. The studies were of fair quality (Table 3).

**Table 3:** Quality assessment of the included studies based on the Newcastle-Ottawa Scale

Study number	Author	Year	Selection Bias Assessment								Comparability		Outcome (Maximum 3 Stars)						Total Score (Maximum 10 Stars)
											(Maximum 2 stars)								
			(Maximum 4 stars)																
																				Representativeness of the exposed cohort		Selection of the non-exposed cohort		Ascertainment of exposure		Demonstration that the outcome of interest was not present at the start of the study		Comparability of cohorts on the basis of the design or analysis		Assessment of the outcome		Was the follow-up long enough for outcomes to occur		Adequacy of follow-up of cohorts	
			Selection	Score	Selection	Score	Selection	Score	Selection	Score	Selection	Score	Selection	Score	Selection	Score	Selection	Score		
			6	Bodem et al. [9]	2015	*						*				*		*		*		5
8	Gaal et al. [11]	2016	*						*				*		*		*		5
10	Davis et al. [13]	2016	*						*				*		*		*		5
11	Roistacher et al. [14]	2021	*				*		*				*		*		*		6

The certainty of evidence for each outcome was evaluated using the Grading of Recommendations, Assessment, Development, and Evaluation (GRADE) approach, considering five domains: risk of bias, inconsistency, indirectness, imprecision, and publication bias. The overall certainty of evidence ranged from low to moderate across outcomes (Table 4). Evidence supporting the use of antibiotic prophylaxis for reducing SSIs was rated as low-certainty, mainly due to methodological limitations in several randomized controlled trials, heterogeneity in surgical procedures, and variability in antibiotic regimens. For implant survival, evidence was rated as moderate-certainty, with consistent findings indicating a small reduction in early implant failure among patients receiving preoperative antibiotics. Evidence on postoperative pain and inflammation was of low certainty, reflecting inconsistency between studies and indirectness of outcomes.

**Table 4:** Summary of findings table (Cochrane GRADE Approach) Antibiotic prophylaxis compared to no prophylaxis in maxillofacial surgery. The certainty of the evidence was assessed using the GRADE approach across five domains: risk of bias, inconsistency, indirectness, imprecision, and publication bias. GRADE: Grading of Recommendations, Assessment, Development, and Evaluation; RCTs: randomized controlled trials; RR: relative risk

Outcome	Number of Participants (Studies)	Relative Effect (95% CI)	Absolute Effect	Certainty of Evidence (GRADE)	Comments/Reasons for Downgrading
Surgical site infection	~4,000 (10 studies: seven RCTs, three cohorts)	RR ≈ 0.80 (0.60–1.10)	↓ possible 1%–3% absolute risk	⭑⭑◯◯ Low	Downgraded for risk of bias (non-blinded, small samples) and inconsistency (different surgeries/regimens).
Implant failure rate	834 (two RCTs)	RR 0.75 (0.50–0.95)	↓ 3% absolute risk	⭑⭑⭑◯ Moderate	Consistent findings but limited trials; imprecision in confidence interval.
Postoperative pain and inflammation	246 (two RCTs)	Lower mean pain scores (p<0.05 in one study)	↓ mild improvement	⭑⭑◯◯ Low	Downgraded for inconsistency and indirectness.
Adverse drug effects	121 (two RCTs)	No major difference	No significant effect	⭑⭑⭑◯ Moderate	Adequate reporting but small sample size.

Adverse effects and antibiotic resistance were supported by moderate-certainty evidence, with minimal serious adverse events reported but limited by small sample sizes and observational study designs. Overall, while antibiotic prophylaxis may reduce early implant failures, there is insufficient high-certainty evidence to support its routine use across all maxillofacial surgical procedures.

Discussion

There doesn't seem to be any additional clinical advantage to patients with maxillofacial fractures receiving postoperative prophylactic antibiotic medication in terms of SSI prevention. When we categorized our results according to the quality of the study, the kind of fracture (mandibular), and the surgical technique (open reduction), this conclusion remained valid. Due to inconsistent trial reporting, we were unable to draw any strong findings regarding the dangers associated with prescriptions for postoperative preventive antibiotic therapy.

When contrasted to a placebo, prophylactic administration of antibiotics could reduce early implant failure in healthy patients; however, prophylactic administration and other regimens were not compared. They were of the opinion that short-duration antibiotic treatment, such as as much as two or three grams of amoxicillin an hour before the implant placement process, or 1 g of amoxicillin one hour prior to the implant placement procedure, followed by 500 mg four times a day for two days after surgery, might substantially decrease the rate of early implant failure.

The objective of this study was to evaluate the effects of prophylactic preoperative antibiotics on dental implant osseointegration, postoperative pain, morbidity, and disruption of daily activities. It is still debatable whether prophylactic antibiotics should be used during dental implant surgery. As described by Nolan et al. (2014), a 2008 paper by an ad hoc committee titled "Guidelines for the Provision of Dental Implants" appeared in the International Journal of Oral and Maxillofacial Implants and did not address preventive antibiotic use. The results of the study indicated that, in comparison to not using any antibiotics at all, preoperative prophylactic use of antibiotics may improve dental implant survival [6].

Oral and maxillofacial surgery procedures that are frequently performed include orthognathic surgery. Patients requiring surgical relocation of the maxilla or mandible due to significant dentofacial abnormalities are targeted for this procedure in order to establish optimal occlusal function and esthetic facial harmony. Although this procedure is considered "clean contaminated," one of the most often reported consequences is the rate of postoperative infection, which is a major cause for concern [18]. Antibiotics used during perioperative care are frequently utilized to reduce infection rates. During the initial phase of anesthesia, each patient in this trial received a single intravenous infusion of 1 g of amoxicillin clavulanate. Included in the β-lactam antibiotic class, amoxicillin clavulanate is widely recognized for its ability to combat oral and nasal bacteria, which are the primary impurities in orthognathic surgery. Antibiotic prophylaxis during orthognathic operations has been shown in numerous published studies to lower the risk of infection; nevertheless, there is still debate regarding the best antibiotic to use and how long to administer it for. The authors' prior research indicates that for the purpose of preventing infection following bilateral sagittal split osteotomy surgeries, it is better to take antibiotics for a short period of time after surgery rather than just once before [15].

The reduction in bacterial contamination of the surgical wound may account for the correlation between the administration of antibiotics and the amelioration of postoperative complications. Consequently, inflammatory mediators would be reduced, lowering surgical discomfort without necessarily changing the percentage of postoperative infections [7]. One of the RCT's findings demonstrates that using antibiotics to prevent problems after surgery is supported; there is no benefit to administering them prior to or following surgery versus waiting until after it is finished. To determine if routine antibiotic therapy is warranted, it is necessary to assess whether postoperative sequelae are severe and frequent enough. To be able to prevent the adverse effects of antibiotics, such as the development of bacterial resistance, other measures must also be taken into account, such as the application of local antibiotics. The placebo group experiences more side effects, most likely as a result of the rescue analgesics given to those individuals due to their elevated pain and inflammation [8].

This analysis has certain limitations. Considerable heterogeneity existed among studies in terms of surgical procedures, antibiotic regimens, and outcome measures. Many trials had small sample sizes, inconsistent reporting, and limited follow-up, restricting strong conclusions. Differences in study quality, patient characteristics, and perioperative protocols may have influenced results. Moreover, potential publication bias and inadequate reporting of adverse effects further limit the reliability of the findings.

## Conclusions

Single-dose prophylactic antibiotics provide satisfactory implant survival while reducing cost and resistance risk, though larger trials are needed to set protocols. They may reduce pain and swelling in third molar surgery but do not prevent infections and carry higher side-effect risks. In implant surgery, benefits are mainly for complex or prolonged cases, with no added value for routine single implants.
